# Combined antibiotic therapy spacers either commercial or handmade are superior to monotherapy – a microbiological analysis at the second stage of revision

**DOI:** 10.5194/jbji-6-305-2021

**Published:** 2021-08-10

**Authors:** André Dias Carvalho, Ana Ribau, Daniel Soares, Ana Claudia Santos, Miguel Abreu, Ricardo Sousa

**Affiliations:** 1 Department of Orthopaedics, Centro Hospitalar Universitário do Porto, Porto, Portugal; 2 Department of Microbiology, Centro Hospitalar Universitário do Porto, Porto, Portugal; 3 Department of Infectious Diseases, Centro Hospitalar Universitário do Porto, Porto, Portugal; 4 Porto Bone and Joint Infection Group (GRIP), Centro Hospitalar Universitário do Porto and Grupo TrofaSaude – Hospitais, Portugal

## Abstract

**Background:** Antibiotic-loaded spacers are often used during two-stage exchange for periprosthetic joint infections (PJIs) both for its mechanical properties and as a means of local antibiotic delivery. **Purpose:** The main goal of this study is to compare the efficacy of different options of antibiotic(s) in spacers concerning the rate of positive cultures at the second stage. **Patients and Methods:** We retrospectively evaluated two-stage exchange procedures for infected hip or knee arthroplasty performed between 2012 and 2018 in which adequate (at least four deep tissue samples) culture results in both stages were available. The type of spacer and antibiotics used, in addition to several other patient, infection and treatment-related variables, were registered and correlated to microbiological findings in the second stage. **Results:** Fifty-eight cases were included with a 19.0 % (11/58) overall rate of positive cultures during reimplantation. With a mean follow-up of 46 months, failure rate was significantly higher at 63.6 % (7/11) in cases with positive cultures at reimplantation compared to 4.3 % (2/47) for those with negative cultures during reimplantation (p< 0.001). The need for additional surgeries was also significantly higher (odds ratio (OR) 122.67, confidence interval (CI) 95 % 11.30–1331.32, p< 0.001). Multivariable analysis revealed antibiotics in the spacers were the main independent prognostic risk factor associated with positive cultures at the second stage with an advantage for combined antibiotics. Monotherapy is associated with failure with an OR of 16.99. Longer time between surgeries did not have statistical significance (p= 0.05), and previous surgical treatment for PJI, presence of difficult-to-treat microorganism(s), duration of systemic antibiotic therapy or even treatment within a dedicated septic team were not shown to be independent risk factors. Among combined antibiotic spacers, there were no significant differences between the rate of positive cultures during the second stage, comparing commercially available vancomycin/gentamicin spacers to hand-mixed vancomycin/meropenem manufactured spacers (8.3 % [2/24] vs. 15.0 % [3/20], p= 0.68). **Conclusions:** Results show that combined antibiotic therapy spacers are advantageous when compared to gentamicin monotherapy as they produce significantly lower rates of subsequent positive cultures during the second stage. Hand-mixed high-dose vancomycin/meropenem spacers seem to perform just as well as prefabricated commercially available vancomycin/gentamicin options. **Level of Evidence:** Therapeutic level III.

## 1 Introduction

Periprosthetic joint infection (PJI) is a feared complication after total joint arthroplasty. It is indisputably a source of significant deleterious impact on a patient's health status and quality of life. As the requirements for total joint arthroplasty have been increasing and are expected to continue to steadily increase so will the burden of infection (Patel et al., 2015).

PJI treatment options are greatly limited by the presence of bacterial biofilm on the prosthesis' surface. These are highly structured communities of microbial cells surrounded by an extracellular matrix that protects them against the host's immune system action and virtually every antibiotic (Tzeng et al., 2015). Once a mature biofilm is established, removing the implant is the only sensible alternative to eradicate the infection. As such, exchange revision surgery is widely considered to be the gold standard treatment for PJI.

Although one-stage exchange is gaining momentum worldwide, it is mostly used in selected cases, and two-stage revision surgery is still the most common alternative (Leite et al., 2016). When a two-stage strategy is preferred, most surgeons agree on the use of antibiotic-loaded cement spacers with two main objectives in mind: local antibiotic delivery and preservation of joint stability and function between stages that offers some comfort to the patient and prevents soft-tissue contractures. Ultimately, they contribute to an easier and faster second-stage reimplantation surgery (Marczak et al., 2016; Nahhas et al., 2020).

Several different spacer options exist. They can be static or mobile and prefabricated or handmade, and a number of different antibiotics can be incorporated. Despite the large amount of research on this topic, several controversies persist, and it is not clear which (if any) is the best choice. The main goal of this paper is to, based on the rate of positive cultures at the second stage, compare the efficacy of single vs. combined antibiotics in eradicating PJI. A secondary goal is to ascertain a possible performance difference between prefabricated and handmade spacers.

## 2 Material and methods

We retrospectively evaluated all two-stage exchange procedures performed at our university hospital for infected hip or knee arthroplasty between 2012 and 2018. Basic patient demographics and comorbidities were registered, as well as infection-related clinical variables such as type of implant (primary or revision prosthesis), whether there were previous surgeries for infection, and microbiological findings in the first and second stages. Methicillin-resistant *Staphylococcus aureus*, Gram-negative rods, *Enterococcus* species and fungi were further classified as difficult-to-treat microorganisms.

Treatment-related information such as duration of first-stage surgery, the type (commercially available or handmade) and antibiotic(s) used in the spacer, time interval between stages, duration of systemic antibiotic therapy and whether the patient was operated on by a dedicated septic team were also thoroughly collected. Cases in which no cultures were obtained during reimplantation and cases without sufficient data on antibiotic(s) used in cement spacers were excluded.

### 2.1 Definitions

Definitive diagnosis of PJI during the first stage was based on the diagnostic criteria proposed back in 2011 by the Musculoskeletal Infection Society (Parvizi et al., 2011) and further refined in 2013 (Gehrke and Parvizi, 2013). Given the lack of accuracy of aforementioned criteria to identify persistent infection at the second stage (George et al., 2016), a different criterion was adopted. Reimplantation cultures were considered significant when (i) at least one of the virulent microorganisms was isolated (*Staphylococcus aureus*, Gram-negative rods, *Enterococcus* species, *Streptococcus* species or *Candida* species) or (ii) at least two of the low-virulence microorganisms with the same antibiogram were isolated (coagulase-negative staphylococci, *Corynebacterium* species, *Cutibacterium acnes* or other).

Failure after reimplantation was defined as the need for additional surgical intervention and/or the need for suppressive antibiotic therapy due to persistent clinical signs of infection.

### 2.2 Surgical and microbiology sampling protocol

During the first stage, no perioperative antibiotic prophylaxis is generally used. Institutional guidelines are to start broad-spectrum antibiotics (vancomycin and piperacillin/tazobactam) after deep tissue sampling (favouring the bone-implant interface) and prosthesis removal for sonication. The choice of which specific type of spacer to use was at the surgeon's personal discretion based on availability and specific considerations in each case.

Systemic antibiotic therapy was prescribed based on antibiotic sensitivity profile of isolated microorganisms. The timing of reimplantation was decided by treating physicians and was mostly based on trending serum inflammatory parameters and clinical impression in each case.

Second-stage surgery is routinely performed under cefazolin prophylaxis, followed by a broad-spectrum antibiotic regimen despite initial microbiology results that are continued until intraoperative culture results. At least four tissue samples (or three tissue samples and sonication of the removed implant and/or spacer) were required to classify them as adequate sampling in either the first or the second stage.

All patients who had positive cultures at reimplantation were subsequently treated with appropriate antibiotics for an additional 12 to 24 weeks.

### 2.3 Statistical analysis

IBM SPSS Statistics 24 was used for the statistical analysis. The groups were compared using the Student's t test (quantitative variable) and Fisher or chi-squared test (qualitative variables). All tests were run using two tails with a significance level set at p< 0.05. Variables that demonstrate a difference in the univariate analysis with p≤0.2 were included in a binomial logistic regression.

## 3 Results

Medical charts of 81 two-stage exchange total hip arthroplasty (THA) or total knee arthroplasty (TKA) cases were reviewed. Thirteen cases were excluded because of inadequate cultures obtained during second-stage surgery, and 10 cases were excluded due to a lack of reliable information on the antibiotic(s) used in the cement spacer. Demographic and clinical information of the 58 patients ultimately included in the final analysis are shown in Table 1.

**Table 1:** Demographic and clinical information of the 58 patients included.

Age (years)${}^{1}$	67.2 (39–85)
Female gender (%)	32 (55.2 %)
ASA classification ≥ 3	29 (50 %)
BMI ≥ 30	20 (34.5 %)
Diabetes mellitus	26 (44.8 %)
Hip : knee ratio	22 : 36
Primary : revision prosthesis	41 : 17
Previous surgical treatment for PJI (%)	
– None	30 (51.7 %)
– DAIR	22 (37.9 %)
– Revision surgery	6 (10.4 %)
Difficult-to-treat microorganism(s) (%)	15 (25.9 %)
Duration of first stage (min)${}^{2}$	162 (± 50)
Antibiotics in spacer (%)	
– Monotherapy	14 (24.1 %)
– Combined	44 (75.9 %)
Duration of systemic antibiotics (days)${}^{1}$	70 (42–171)
Time between surgeries (days)${}^{1}$	143 (56–524)

${}^{1}$ expressed as mean (range); ${}^{2}$ expressed as mean (± standard deviation); ASA – American Society of Anesthesiologists; BMI – body mass index; PJI – periprosthetic joint infection; DAIR – debridement, antibiotics and implant retention.

The majority of cases had undergone previous surgical treatment for PJI, mostly failed previous debridement and implant retention but also failed previous two-stage exchange.

Concerning type of spacer, prefabricated commercially available gentamicin-loaded spacers were applied in 14 cases (24.1 %), and gentamicin- and vancomycin-loaded spacers were used in 24 cases (41.4 %). Hand-mixed high-dose vancomycin (3 g) plus meropenem (2 g) per 40 g of low-dose gentamicin (0.5 g) polymethyl methacrylate (PMMA) manufactured spacers were used in 20 cases (34.5 %). Dual antibiotic therapy spacers were less often used in THA than TKA (54.5 % [12/22] vs. 88.9 % [32/36]), p<0.001). At the beginning of the study (2012), at our institution, we used a specific commercial hip spacer with very good mechanical characteristics but with only one antibiotic (gentamicin).

Microbiological findings in the first stage are summarized in Table 2. PJI cases during the first stage categorized as difficult-to-treat microorganism included three cases of methicillin-resistant *Staphylococcus aureus*, two including *Enterococcus* species, six cases involving Gram-negative rods, one fungi case and three polymicrobial infections including any of the previous bacteria.

**Table 2:** Microorganisms isolated in the first stage of the 58 patients included.

Microorganism(s)	Overall (n=67)
Gram positive	56 (83.6 %)
*Staphylococcus aureus* (SA)	18 (26.9 %)
MRSA	4 (6.0 %)
MSSA	14 (20.9 %)
CoN Staphylococci (S)	25 (37.3 %)
MR CoNS	7 (10.4 %)
MS CoNS	18 (26.9 %)
Other Gram positive	13 (19.4 %)
*Streptococcus* species	9 (13.4 %)
*Enterococcus* species	3 (4.5 %)
*Corynebacterium* species	1 (1.5 %)
Gram negative	10 (14.9 %)
*Enterobacteriaceae*	6 (9.0 %)
*Escherichia coli*	2 (3.0 %)
*Klebsiella* species	2 (3.0 %)
*Proteus* species	1 (1.5 %)
*Pantoea* species	1 (1.5 %)
*Pseudomonas* species	4 (5.9 %)
Fungi	1 (1.5 %)
*Candida albicans*	1 (1.5 %)
Polymicrobial${}^{*}$	12 (20.7 %)

MR – methicillin-resistant; MS – methicillin-sensitive; CoN – coagulase negative; ${}^{*}$ refers to number of polymicrobial PJI cases; specific microorganisms involved are reflected under their respective categories.

**Table 3:** Analysis of risk factors for positive cultures in the second stage in all patients.

	Positive	Negative						
	cultures	cultures	Univariate analysis			Multivariable analysis		
	(n= 11)	(n= 47)	P value	Odds ratio	95 % CI	P value	Odds ratio	95 % CI
Age (years)${}^{1}$	66.5	67.3	0.78	–	–	–	–	–
	(52–79)	(39–85)						
Female gender (%)	8	24	0.31	–	–	–	–	–
	(72.7 %)	(51.1 %)						
ASA classification ≥ 3 (%)	7	22	0.72	–	–	–	–	–
	(63.3 %)	(46.8 %)						
BMI ≥ 30 (%)	3	17	0.73	–	–	–	–	–
	(27.2 %)	(36.2)						
Diabetes mellitus	3	23	0.31	–	–	–	–	–
	(27.2 %)	(48.9)						
Revision prosthesis (%)	4	13	0.71	–	–	–	–	–
	(36.4 %)	(27.7 %)						
Hip location (%)	7	15	0.05	3.73	(0.95–14.74)	0.42	4.37	(0.12–153.39)
	(63.6 %)	(31.9 %)						
Previous surgical treatment								
for PJI (%)								
– Yes	8 (72.7 %)	20 (42.6 %)	0.07	3.60	(0.85–15.31)	0.17	47.15	(2.24–993.76)
– No	3 (27.3 %)	27 (57.4 %)						
Difficult-to-treat	5	10	0.13	3.08	(0.79–12.22)	0.14	0.20	(0.02–1.74)
microorganism(s) (%)	(45.5 %)	(23.4 %)						
Duration of first stage	1	13	0.27	–	–	–	–	–
> 75th percentile (%)	(9.1 %)	(27.7 %)						
Antibiotics in spacer (%)								
– Monotherapy	6 (54.5 %)	8 (17.0 %)	0.02	0.20	(0.05–0.79)	0.03	16.99	(1.87–901.83)
– Combined	5 (45.5 %)	39 (83.0 %)						
Dedicated septic team (%)	3 (27.3 %)	28 (59.6 %)	0.05	3.93	(0.92–16.74)	0.43	–	–
Duration of systemic	93 (± 51)	64 (± 28)	0.09	–	–	0.26	–	–
antibiotics (days)${}^{2}$								
Time between surgeries	220	125	0.03	–	–	0.05	–	–
(days)${}^{2}$	(± 121)	(± 79)						

${}^{1}$ expressed as mean (range); ${}^{2}$ expressed as mean (± standard deviation); ASA – American Society of Anesthesiologists; BMI – body mass index; PJI – periprosthetic joint infection.

There was significant growth in cultures taken during the second stage in 11 (19.0 %) cases. Failure rate after reimplantation was 15.5 % (9/58) with a mean follow-up of 46 months (interquartile range 24–48 months) after the second stage. It was significantly higher in those patients who had positive cultures during the second stage (63.6 % [7/11]) compared to those with negative cultures (4.3 % [2/47]). Despite the fact that all positive cases were subsequently treated with systemic antibiotics for a period of 12 to 24 weeks (OR 2.5, CI 95 % 1.26–3.80, p< 0.001). The likelihood of additional surgeries was significantly higher (OR 122.67, CI 95 % 11.30–1331.32, p<0.001) in this subgroup of patients. Variables possibly associated with positive cultures during the second stage in the univariate analysis were hip location, antibiotic monotherapy in spacer, treatment performed by a non-dedicated septic team and longer time between first and second stage (Table 3).

**Table 4:** Analysis of risk factors for positive cultures in the second-stage surgery among patients with combined antibiotics in spacer.

	Overall	Positive cultures	Negative cultures	
	(n= 44)	(n= 5)	(n= 39)	P value
Age (years)${}^{1}$	67.2 (39–85)	68.8 (61–75)	68.7 (39–85)	1.00
Female gender (%)	26 (59.1 %)	5 (100 %)	21 (53.8 %)	0.06
ASA classification ≥ 3 (%)	22 (50 %)	3 (60.0 %)	19 (48.7 %)	1.00
BMI > 30 (%)	18 (40.1 %)	2 (40.0 %)	16 (41.0 %)	1.00
Diabetes mellitus	21 (47.7 %)	2 (40.0 %)	19 (48.7 %)	0.35
Revision prosthesis (%)	12 (27.3 %)	2 (40.0 %)	10 (25.6 %)	1.00
Hip location (%)	12 (27.3 %)	3 (60.0 %)	9 (23.1 %)	0.12
Previous surgical treatment for PJI (%)				
– Yes	20 (45.5 %)	4 (80.0 %)	16 (41.0 %)	0.65
– No	24 (54.5 %)	1 (20.0 %)	23 (59.0 %)	
Difficult-to-treat microorganism(s) (%)	12 (27.3 %)	2 (40.0 %)	10 (25.6 %)	0.59
Duration of first stage > 75th percentile (%)	11 (25 %)	2 (40.0 %)	9 (23.1 %)	1.00
Dedicated septic team (%)	30 (68.1 %)	3 (60.0 %)	27 (69.2 %)	0.64
Duration of systemic antibiotics (days)${}^{2}$	60 (42–171)	59 (± 26)	68 (± 47)	1.00
Time between surgeries (days)${}^{2}$	121 (56-392)	112 (± 75)	192 (± 90)	0.35

${}^{1}$ Expressed as mean (range); ${}^{2}$ expressed as mean (± standard deviation); ASA – American Society of Anesthesiologists; BMI – body mass index; PJI – periprosthetic joint infection.

Multivariable analysis, performed including variables with p≤0.2, substantiates gentamicin monotherapy in spacer (OR 16.99;, 95 % CI 1.87–901.83, p= 0.03) as the only significant independent prognostic factor for positive cultures in the second stage. Longer time between surgeries appears to be associated with higher probability of positive cultures in the second stage, although it does not quite reach statistical significance (p= 0.05). Previous surgical treatment for PJI, revision prosthesis, patient comorbidities, presence of difficult-to-treat microorganism(s), duration of systemic antibiotic therapy or even treatment within a dedicated septic team did not emerge as independent risk factors.

Among patients receiving combined antibiotic therapy spacers, no other registered variable appeared as a significant risk factor (Table 4).

Further analysis exploring combined antibiotic therapy spacers found no statistically significant difference between the gentamicin- and vancomycin-loaded prefabricated commercially available spacers and the high-dose vancomycin and meropenem hand-mixed manufactured spacers (8.3 % [2/24] vs. 15.0 % [3/20], p= 0.68). Despite the rate of positive cultures during the second stage, we compared the complication rate between commercial spacers and hand-mixed manufactured spacers. The overall rate of complications was 8.6 % (5/58) (3 dislocations of hip spacers and 2 fractures) and there is no difference between the two types of spacers.

**Table 5:** Microbiological findings at the first stage and type of spacer used in those cases with positive culture cases at the second stage.

	Antibiotic(s)	Microorganism(s)	Sensitivity${}^{*}$ to	Microorganism(s)	Sensitivity${}^{*}$ to	Microorganism at
	in spacer	at first stage	antibiotics in	at second stage	antibiotics in	first vs. second
			spacer		spacer	stage
1	Gentamicin	*Staphylococcus aureusStreptococcus gordonii*	Sensible	*Staphylococcus aureus*	Sensible	Likely persistent
2	Gentamicin	*Pseudomonas aeruginosaStaphylococcus aureus*	Resistant	*Staphylococcus aureus*	Resistant	Likely persistent
3	Gentamicin	*Staphylococcus aureus*	Resistant	*Staphylococcus aureus*	Resistant	Likely persistent
4	Gentamicin +vancomycin	*Staphylococcus lugdunensis*	Sensible	*Enterococcus faecalis*	Resistant	Different
5	Gentamicin +vancomycin	*Streptococcus sanguinisStaphylococcus aureus*	Sensible	*Staphylococcus aureus*	Sensible	Likely persistent
6	Vancomycin +carbapenem (+ gentamicin)	*Staphylococcus aureus*	Sensible	*Staphylococcus epidermidisStaphylococcus capitis*	Sensible	Different
7	Gentamicin	*Staphylococcus aureus*	Sensible	*Staphylococcus aureus*	Sensible	Likely persistent
8	Gentamicin +vancomycin	*Staphylococcus epidermidisCorynebacterium striatumCandida albicans*	Resistant	*Candida albicans*	Resistant	Likely persistent
9	Gentamicin	*Staphylococcus epidermidisStaphylococcus haemolyticusCorynebacterium striatum*	Resistant	*Corynebacterium striatum*	Resistant	Likely persistent
10	Gentamicin	*Klebsiella pneumoniaeStaphylococcus epidermidis*	Resistant	*Klebsiella pneumoniae*	Resistant	Likely persistent
11	Vancomycin +carbapenem (+ gentamicin)	*Staphylococcus caprae*	Sensible	*Staphylococcus capraeProteus mirabilis*	Sensible	Likely persistent

When focusing on the influence of the type of microorganisms, we found no significant difference of the presence of classically considered difficult-to-treat bacteria. We found that 81.8 % (9/11) of microorganisms isolated during second-stage cultures were some kind of persistent microorganism already present in the first stage (Table 5). As mentioned above, systemic antibiotic therapy after first stage was prescribed based on antibiotic sensitivity profile of isolated microorganism, and was never less than 6 weeks. Timing of reimplantation was decided by treating physician(s) and was mostly based on trending serum inflammatory parameters and clinical impression in each case.

When the microorganisms isolated in the first stage are resistant to antibiotics in the spacer, the risk of positive cultures on reimplantation is significantly higher despite appropriate systemic antibiotic therapy between stages (OR 18.75, 95 % CI 2.954–118.99, p= 0.002). More detailed information on the 11 cases with positive cultures during the second stage can be found on Table 4. Naturally, finding persistent resistant microorganisms is more frequent when using monotherapy spacers (four out of six) than in dual antibiotic spacers (one out of five). Of the 11 cases with positive cultures, four patients were treated with another two-stage revision, four with suppressive antibiotherapy and three with a DAIR procedure. The four patients under suppressive antibiotherapy, initially, start broad-spectrum antibiotics (vancomycin and piperacillin/tazobactam), and after the microbiological results (7–10 d) they switch to antibiotic sensitivity profile of isolated microorganisms (including one antibiofilm antibiotic).

## 4 Discussion

Periprosthetic joint infection (PJI) treatment is complex and laborious. Although several controversies still exist, there is a consensus that chronic infections, with established and mature biofilm, require complete exchange of the implant. Currently, the most popular strategy is still to perform one initial surgery to accomplish exhaustive surgical debridement and complete implant removal followed by a second surgery in a few weeks to reimplant a new prosthesis after the infection is deemed to be cured, i.e. a two-stage exchange. When this two-stage strategy is preferred, the goal of the interim period between surgeries is to eradicate infection while optimizing local conditions for a successful revision surgery.

A major potential advantage of the use of spacers is the possibility of local antibiotic therapy. The local antibiotic delivery theoretical advantage is that it can result in very high drugs concentration at the site of infection with low serum levels, thus reducing its potential toxicity (Osmon et al., 2013; Cui et al., 2007).

Although some authors advocate that the choice of antibiotics needs to be individualized for each case based on the pathogen antibiotic susceptibility, often there is no pathogen identified preoperatively, and in addition it has been shown that a significant proportion of cases will show disagreement between pre- and intra-operative microbiological findings (Holleyman et al., 2016; Matter-Parrat et al., 2017).

As such, our approach has been to adopt broad-spectrum antibiotics in spacers. Traditionally, available commercial spacers are impregnated with gentamicin which has a good activity against a wide range of Gram-positive and Gram-negative bacteria. However, the high prevalence of gentamicin resistance especially among coagulase-negative staphylococci fuelled our search for an alternative (Lourtet-Hascoet et al., 2018). Given the characteristics of PJI bacterial flora in our institution (Sousa et al., 2010), we decided that we would like to have broad Gram-positive coverage, and that led us to adopt the use of surgeon hand-mixed cement spacers incorporating vancomycin. Aminoglycosides for Gram-negative coverage would be a natural association with vancomycin but a lack of available powder formulation for cement mixing in our pharmacy led us to the search of an adequate alternative, and meropenem was ultimately chosen (Soares et al., 2015). Our policy over the last few years has been to hand mix 3 g of vancomycin and 2 g of meropenem for each 40 g of gentamicin-loaded PMMA both for knee and hip spacers. More recently, commercial prefabricated spacers containing both vancomycin and gentamicin have become available and were also incorporated into our clinical practice, especially when long-stem hip spacers are required. This has created a “natural experiment” setting to evaluate the performance of these different spacers.

During the second-stage surgery, a new debridement is performed, and multiple deep tissue samples are routinely sent for microbiology. We believe that using these results is an adequate endpoint to assess antibiotic therapy effectiveness, specifically spacer antibiotics. It has been shown that positive cultures correlate with increased risk of reinfection, and a direct correlation seems to exist between higher bacterial load and subsequent risk of failure (Nelson et al., 2014; Sorli et al., 2012; Wouthuyzen-Bakker et al., 2019; Akgun et al., 2017). Current results confirm a much higher risk of subsequent failure when second-stage cultures show significant growth.

With this outcome in mind, our results show the most important prognostic factor to be the use of combined (dual or triple) antibiotic therapy spacers. The use of gentamicin monotherapy spacers is independently associated with much higher risk of positive cultures in the second stage. Although others have suggested positive cultures in the second stage are mostly caused by microorganisms other than the ones isolated during the first stage (Wouthuyzen-Bakker et al., 2019; Akgun et al., 2017), this was not the case in our study where the majority of positive cultures in the second stage revealed some kind of persistent microorganism already present in the first stage. Naturally, it is more common to encounter gentamicin-resistant microorganisms that persist than to encounter infections with microorganisms resistant to both vancomycin and gentamicin or meropenem. Despite the large number of variables that may influence this outcome (systemic antibiotic therapy, quality of initial surgical debridement, etc.), the fact that this effect persisted after multivariable analysis suggests it is a truly independent prognostic factor.

Our secondary goal was to ascertain a possible performance difference between prefabricated and handmade spacers. Surgeon-made spacers by manually adding and mixing antibiotics are often criticized not for their mechanical or functional properties but rather for their erratic and unpredictable antibiotic release kinetics and subsequent clinical outcome. Nevertheless it has been shown in vitro that high-dose manual addition (> 5 g for every 40 g PMMA) leads to higher antibiotic release compared to prefabricated spacers (Goltzer et al., 2015). Our findings suggest that the chosen combination and dosage of antibiotics for handmade spacers can be just as effective as dual antibiotic commercially available spacers.

Several limitations can be pointed to in the current study. Although systemic antibiotic therapy was decided in each case after knowing antibiotic susceptibilities of the infecting pathogens, there was a myriad of different options, and it was not possible to further explore the influence of each regimen in the outcome. In any case, local antibiotic therapy within the spacer seems to be a more important feature than systemic antibiotic therapy. Also, the retrospective nature of this study and its limited sample size did not allow for stratification of results per type of microorganism, but we did find that the presence of classically difficult-to-treat microorganism does not seem to be a significant factor if they are sensitive to the antibiotics used in the spacer. Although microorganism species and antibiotic susceptibility profile were used to judge the bacteria found in the second stage and judge it as persistent or new, we did not perform genome analysis, and this is but a crude assumption. It is not impossible that some of them are new infections rather than persistent ones.

All facts considered, combined antibiotic therapy spacers seem to be a better alternative in treating periprosthetic joint infections as they result in significantly lower rates of positive cultures taken in the second-stage surgery and lower risk of subsequent failure. Both prefabricated commercially available and surgeon-made antibiotic hand-mixed spacers seem to be valid options as there was no significant performance difference between them.

## Data Availability

In this paper, we worked with the data that is published (in tables); we did not use any other data to make the statistical analysis or get the results. The data used to support the findings of this study are included in the article.
